# Facile Synthesis of Polypyrrole/MnO_2_/Carbon Cloth Composites for Supercapacitor Electrodes

**DOI:** 10.3390/nano15090641

**Published:** 2025-04-23

**Authors:** Yan Chen, Hanyue He, Min Liu, He Xu, Haibo Zhang, Xinghua Zhu, Dingyu Yang

**Affiliations:** 1 College of Optoelectronic Technology, Chengdu University of Information Technology, Chengdu 610225, China; hhycuit@outlook.com (H.H.); lmcuit@outlook.com (M.L.); xuhecuit@outlook.com (H.X.); zhanghaibo@cuit.edu.cn (H.Z.); 2 Intelligent Manufacturing Industry Technology Research Institute, Sichuan University of Arts and Science, Dazhou 635000, China; 3 Dazhou Industrial Technology Research Institute, Dazhou 635000, China; zhuxinghua@xhu.edu.cn; 4 School of Materials Science and Engineering, Xihua University, Chengdu 610039, China

**Keywords:** PPy/MnO_2_/CC, composite, vapor-phase polymerization, electrochemical performance

## Abstract

In the development of flexible smart electronics, fabricating electrodes with optimized architectures to achieve superior electrochemical performance remains a significant challenge. This study presents a two-step synthesis and characterization of a polypyrrole (PPy)-MnO_2_/carbon cloth (CC) nanocomposite. The MnO_2_/CC substrate was first prepared via the hydrothermal method, followed by uniform PPy coating through vapor-phase polymerization in the presence of an oxidizing agent. Electrochemical measurements revealed substantial enhancement in performance, with the specific capacitance increasing from 123.1 mF/cm^2^ for the MnO_2_/CC composite to 324.5 mF/cm^2^ for the PPy/MnO_2_/CC composite at a current density of 2.5 mA/cm^2^. This remarkable improvement can be attributed to the synergistic effects between the conductive PPy polymer and MnO_2_/CC substrate and the formation of additional ion transport channels facilitated by the PPy coating. This work provides valuable insights for designing high-performance electrode materials and advances the development of composite-based energy storage devices.

## 1. Introduction

Wearable smart electronic devices have found extensive applications in health monitoring systems, flexible displays, and photovoltaic cells, ascribed to their flexibility and compact size advantages [1,2,3]. To power these advanced electronics, the development of novel flexible energy storage devices has become indispensable. Among the various options, supercapacitors have emerged as promising energy storage devices due to their high power density, rapid charging/discharging rates, and excellent cycling stability [4,5,6,7]. The performance of supercapacitors widely depends on three key factors: active materials, flexible substrates, and electrode fabrication processes [3,8]. Among the various materials used for electrodes, transition metal oxides and conducting polymers are invariably employed as electrode materials [9,10]. In particular, MnO_2_ stands out as an attractive candidate because of its high theoretical specific capacitance (up to 1375 F g^−1^), cost-effectiveness, and environmental friendliness [11,12,13,14,15]. However, the inevitable disadvantages of MnO_2_-based electrodes, such as poor electronic conductivity and poor structural stability, make it difficult to reach high performance [13]. To overcome these challenges, researchers have explored composite strategies by combining MnO_2_ with highly conductive materials, including carbon-based materials [16,17] and conducting polymers [18]. Polypyrrole (PPy), a commonly used conducting polymer, is an excellent complementary material for MnO_2_ composites. The synergistic combination of MnO_2_′s high pseudocapacitance and PPy’s superior conductivity can significantly enhance overall electrochemical performance [19]. Recent studies have demonstrated promising results with various MnO_2_/PPy composite architectures. For instance, Bahloul et al. exploited γ-MnO_2_/PPy composites via hydrothermal synthesis, achieving a substantially improved specific capacitance (141.6 F g^−1^) [20]. Sidhu et al. fabricated PPy films embedded with MnO_2_ nanoparticles through electrochemical polymerization, obtaining electrodes with both high capacitance and cycling stability [21]. Shivakumara et al. reported a facile co-precipitation method to prepare MnO_2_/PPy nanocomposites exhibiting exceptional discharge capacitance and cycle life [22]. Some other composites have been reported as well. Yalovega et al. prepared NiO_x_/MWCNT and CuO_x_/MWCNT composites, which showed specific capacities of 149 F/g and 37 F/g at a current density of 1 A/g, respectively [23]. For flexible energy storage applications, the choice of substrate is equally crucial. Ideal substrates should combine high conductivity, flexibility, and large surface area [24,25]. With high conductivity, flexible and mechanical robustness, carbon cloth is an excellent candidate as an electrode substrate [26].

In this work, we designed and fabricated a ternary MnO_2_/polypyrrole/carbon cloth (MnO_2_/PPy/CC) hybrid electrode through a combined hydrothermal and vapor-phase polymerization (VPP) approach. The fabrication process involves the following procedures: (1) hydrothermal growth of MnO_2_ nanorods on carbon cloth to ensure strong anchoring, followed by (2) conformal PPy coating via VPP to enhance overall conductivity. These combination methods are highly compatible with large-scale industrial production while maintaining favorable electrochemical performance in the synthetic composites. Moreover, this hierarchical architecture creates efficient ion transport pathways through synergistic effects between MnO_2_ and PPy, leading to significantly enhanced electrochemical performance.

## 2. Experiment

### 2.1. Pretreatment of Carbon Cloth

Prior to use, the carbon cloth (CC) was ultrasonically cleaned in ethanol and deionized water to remove surface impurities. Subsequently, it was treated with a piranha solution (a 3:7 *v*/*v* mixture of 30% H_2_O_2_ and 98% H_2_SO_4_) at 100 °C for 1 h in a water bath to enhance surface hydrophilicity and active site density. Finally, the cloth was thoroughly rinsed with deionized water and dried for further use.

### 2.2. Synthesis of MnO_2_/Polypyrrole on Carbon Cloth

**Figure 1:**
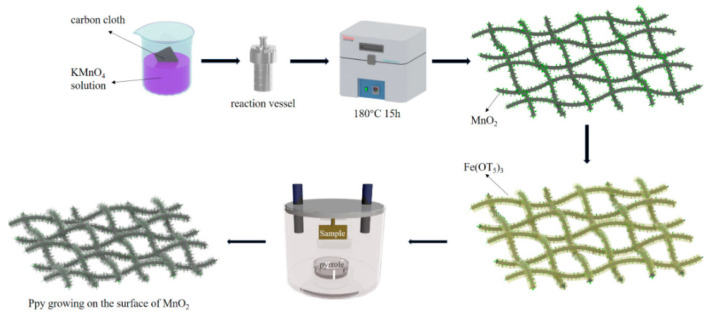
Schematic illustration of the PPy/MnO_2_/CC composite preparation process.

The fabrication process of the MnO_2_/polypyrrole (MnO_2_/PPy) flexible electrode is illustrated in Figure 1.

#### 2.2.1. Hydrothermal Growth of MnO_2_ Nanorods

A homogeneous solution was prepared by dissolving 0.5 g of KMnO_4_ in 40 mL of deionized water under continuous stirring. The pretreated carbon cloth was then immersed in the solution and transferred into a 50 mL Teflon-lined stainless steel autoclave. The sealed autoclave was heated in a muffle furnace at 180 °C for 15 h. After cooling to room temperature, the MnO_2_-coated carbon cloth (MnO_2_/CC) was rinsed repeatedly with deionized water and dried at 60 °C.

#### 2.2.2. Vapor-Phase Polymerization (VPP) of Polypyrrole

To deposit PPy on the MnO_2_/CC substrate, an oxidant solution was first prepared by mixing 8 mL of isopropanol (IPA) with 8 mL of iron(III) p-toluenesulfonate (Fe(OTs)_3_) under ultrasonic agitation for 4 h. The MnO_2_/CC was then dip-coated in this solution to ensure uniform adsorption of the oxidant. After drying, the sample was placed in a vapor-phase polymerization (VPP) chamber and exposed to pyrrole monomer vapor at room temperature, allowing for in situ polymerization and conformal PPy coating.

## 3. Characterization

The crystallographic structures of the synthesized materials were analyzed using X-ray diffraction (XRD, Rigaku SmartLab, Cambridge, UK) and Raman spectroscopy (Advantage, 532 nm). Morphological characterization was performed using field-emission scanning electron microscopy (FE-SEM, Hitachi SU4800, Tokyo, Japan) to examine the surface microstructure of the composite electrodes. Electrochemical measurements were conducted using a CHI660D electrochemical workstation (Chenhua, Shanghai, China) in a standard three-electrode configuration. The working electrode was prepared by cutting the sample to dimensions of 1 cm × 1 cm, while a platinum plate and Ag/AgCl electrode served as the counter and reference electrodes, respectively. All tests were performed in 1 M Na_2_SO_4_ aqueous electrolyte at room temperature. The electrochemical performance of the flexible supercapacitor was evaluated through cyclic voltammetry (CV) and galvanostatic charge–discharge (GCD) measurements.

## 4. Results and Discussion

### 4.1. X-ray Diffraction Analysis

**Figure 2:**
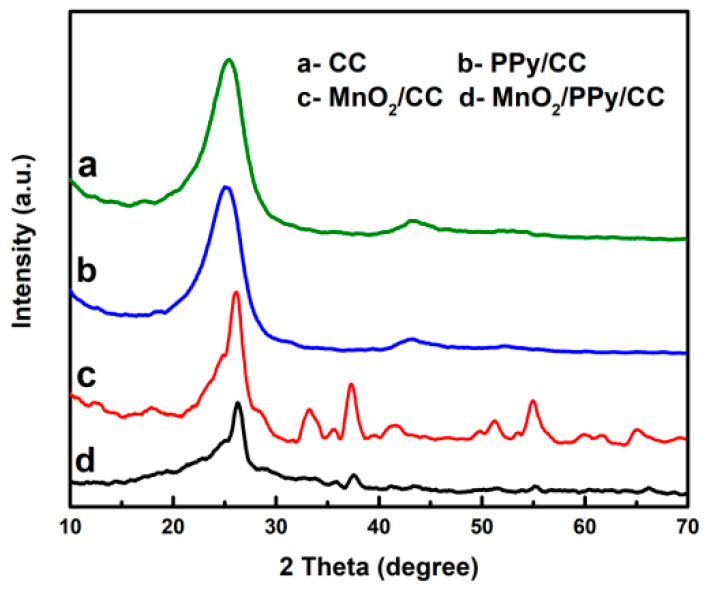
XRD patterns of (**a**) CC, (**b**) PPy/CC, (**c**) MnO_2_/CC, and (**d**) PPy/MnO_2_/CC.

X-ray diffraction (XRD) was employed to characterize the crystalline structures of the synthesized electrodes. Figure 2 displays the XRD patterns of the (a) bare carbon cloth (CC), (b) PPy/CC, (c) MnO_2_/CC, and (d) MnO_2_/PPy/CC composites. The pristine CC substrate (Figure 2a) shows a prominent peak at 26.2°, consistent with the (002) plane of graphitic carbon (JCPDS #75-1621) [27]. In contrast, the PPy/CC composite (Figure 2b) exhibits no discernible crystalline peaks beyond those of CC, indicating the amorphous structure of polypyrrole [28]. For the MnO_2_/CC electrode (Figure 2c), distinct diffraction peaks appear at 12.3°, 17.9°, 28.1°, 37.4°, 49.9°, 60.0°, 65.2°, and 69.5°, matching the (110), (200), (310), (211), (411), (521), (002), and (541) planes of α-MnO_2_ (JCPDS #44-0141), respectively, confirming the formation of crystalline α-MnO_2_ via hydrothermal synthesis. In the ternary MnO_2_/PPy/CC composite (Figure 2d), the major α-MnO_2_ peaks at 12.6°, 28.7°, and 37.2° (indexed to the (110), (310), and (211) planes) remain detectable, while some minor peaks observed in MnO_2_/CC are less pronounced. This attenuation likely results from the partial coverage of MnO_2_ by the PPy layer during vapor-phase polymerization.

### 4.2. Raman Spectroscopy Analysis

**Figure 3:**
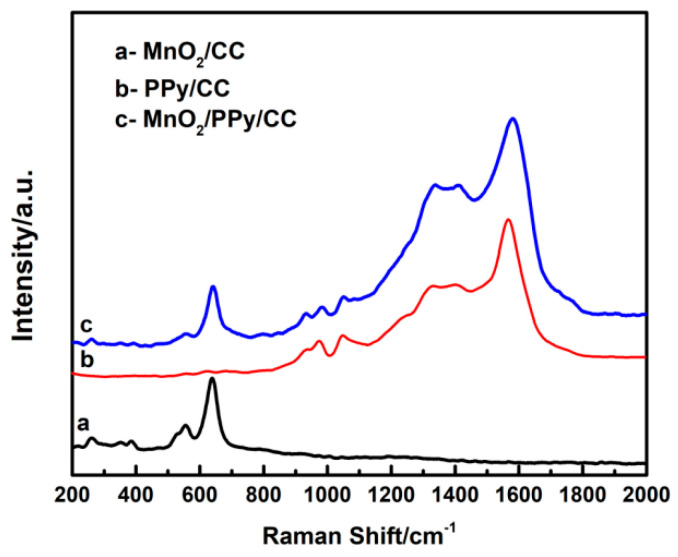
Raman spectra of (**a**) MnO_2_/CC, (**b**) PPy/CC, and (**c**) PPy/MnO_2_/CC.

Raman spectroscopy was utilized to analyze the molecular structure and vibrational properties of the fabricated electrodes. Figure 3 compares the Raman spectra of the (a) MnO_2_/CC, (b) PPy/CC, and (c) MnO_2_/PPy/CC composites. In the MnO_2_/CC spectrum (Figure 3a), a prominent peak appears at 634 cm^−^^1^, assigned to the symmetric Mn–O stretching mode in MnO_6_ octahedra [29], verifying the deposition of manganese dioxide on the carbon substrate. The PPy/CC spectrum (Figure 3b) displays key vibrational bands at 934 and 974 cm^−^^1^ (C–C ring deformation), 1051 cm^−^^1^ (C–H in-plane bending), 1403 cm^−^^1^ (C–N stretching in polaronic PPy), and 1570 cm^−^^1^ (C=C backbone stretching) [30,31,32,33]. For the ternary MnO_2_/PPy/CC composite (Figure 3c), the Raman spectrum exhibits combined features of both MnO_2_ and PPy. The persistence of the Mn–O peak at 634 cm^−^^1^ confirms the structural integrity of MnO_2_, while slight shifts in PPy-related peaks (931, 982, 1047, 1408, and 1575 cm^−^^1^) indicate possible interfacial interactions between MnO_2_ and PPy. The retention of all characteristic bands confirms the successful integration of MnO_2_ and PPy on the carbon cloth via the hybrid hydrothermal and VPP synthesis strategy.

### 4.3. Morphological Characterization via Scanning Electron Microscopy

**Figure 4:**
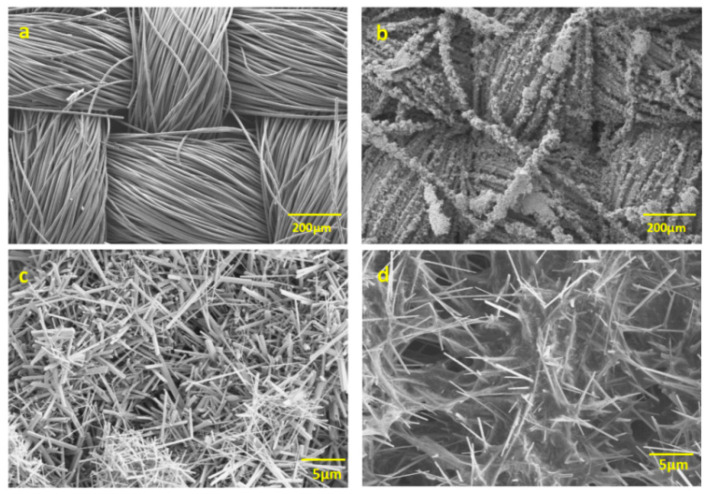
SEM images of (**a**) CC and (**b**) MnO_2_/CC. Higher magnification images of (**c**) MnO_2_/CC and (**d**) PPy/MnO_2_/CC.

The morphological evolution of electrode materials during fabrication was examined via scanning electron microscopy (Figure 4). The bare carbon cloth substrate (Figure 4a) displays a typical woven structure consisting of smooth carbon fibers (∼20 μm diameter). After hydrothermal processing (Figure 4b), the fiber surfaces become uniformly decorated with MnO_2_ nanostructures. High-resolution images (Figure 4c) show these to be densely arranged, acicular MnO_2_ nanorods (∼500 nm diameter) forming an interpenetrating 3D network. This unique architecture introduces multiscale porosity that promotes both high surface area and efficient electrolyte transport [34,35,36]. The ternary composite (Figure 4d) demonstrates successful polypyrrole encapsulation, where the MnO_2_ nanorods appear uniformly coated with a conformal PPy layer. This architecture exhibits advantageous characteristics for supercapacitor applications. The PPy coating enhances electronic conductivity while maintaining ionic accessibility and protects MnO_2_ from structural degradation during cycling. Notably, the vapor-phase polymerization technique achieves complete surface coverage without compromising the nanorod morphology, as evidenced by the maintained structural integrity of the MnO_2_ framework beneath the polymer coating. This morphology is particularly advantageous for electrochemical energy storage, as it combines the pseudocapacitive properties of MnO_2_ with the conductive network of PPy while maximizing the active material–electrolyte contact area.

### 4.4. Electrochemical Performance

**Figure 5:**
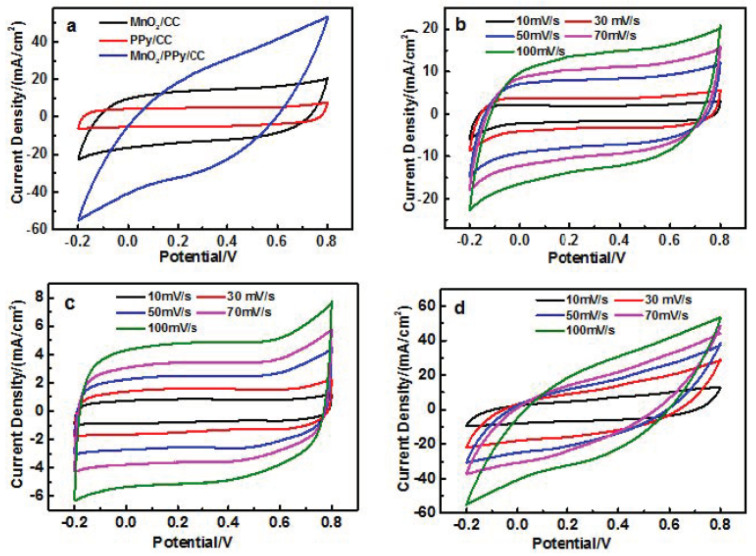
(**a**) CV curves of MnO_2_/CC, PPy/CC, and PPy/MnO_2_/CC electrodes at the scan rate of 100 mV/s; CV curves of (**b**) MnO_2_/CC, (**c**) PPy/CC, and (**d**) PPy/MnO_2_/CC at different scan rates.

Cyclic voltammetry (CV) measurements were conducted to evaluate the electrochemical performance of MnO_2_ and its composite films in 1 M Na_2_SO_4_ electrolyte. Figure 5a presents the CV curves of MnO_2_/CC, PPy/CC, and PPy/MnO_2_/CC at a scan rate of 100 mV/s within a potential window of −0.2 to 0.8 V (vs. Ag/AgCl). All of the samples show a highly symmetrical leaf-like CV shape, which indicates the superior double-layer capacitive performance [37]. The PPy contributes pseudocapacitance through rapid and reversible redox reactions involving charge compensation via ion doping/dedoping. The electrochemical behavior of PPy can be described using the following redox process [38]:

PPy^+^ + e^−^ + A^−^⇔PPy^0^·A^−^ (1)

where [PPy]^+^ represents the oxidized (p-doped) polypyrrole backbone, A^−^ is the charge-balancing anion from the electrolyte, and [PPy]^0^ denotes the neutral (reduced) state. Accordingly, as a key component of the hybrid composite, MnO_2_ provides additional charge storage capacity through reversible redox reactions. The charge storage mechanism in MnO_2_ can be described using the following electrochemical processes [39]:

(MnO_2_)surface + M^n+^ + ne^−^ ⇔ (MnO_2_^n−^M^n+^)surface (2)

MnO_2_ + Na^+^ + e^−^ ⇔ MnOONa (3)

**Figure 6:**
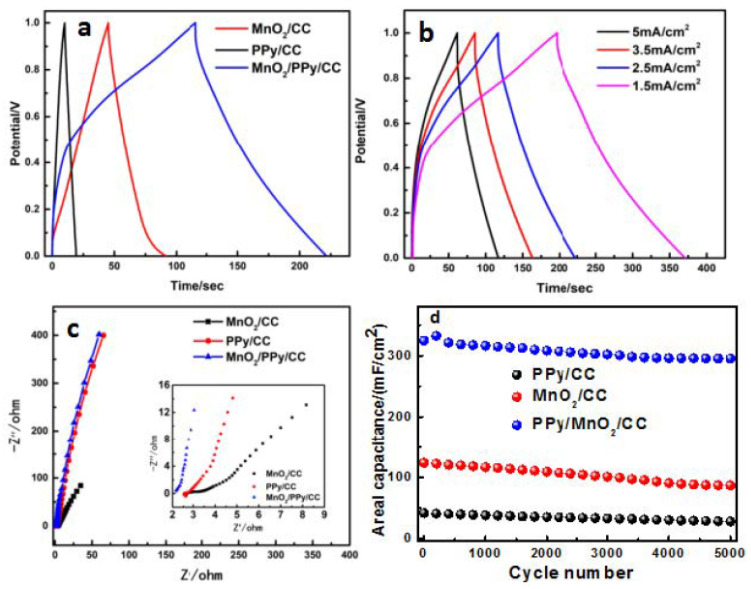
(**a**) GCD curves of the three samples at a current density of 2.5 mA/cm^2^; (**b**) GCD plots of PPy/MnO_2_/CC at 1.5, 2.5, 3.5, and 5 mA/cm^2^; (**c**) the Nyquist plots of impedance data for MnO_2_/CC, PPy/CC, and PPy/MnO_2_/CC in the frequency range of 10^−2^–10^5^ Hz; the inset shows the Nyquist plots of all samples in the high frequency region; (**d**) cycle stability of MnO_2_/CC, PPy/CC, and PPy/MnO_2_/CC at a current density of 2.5 mA/cm^2^.

Notably, the PPy/MnO_2_/CC composite demonstrates the largest integrated CV area among the three samples at identical scan rates, indicating superior capacitance [40]. This enhancement can be attributed to the hierarchical porous structure and synergistic effects between PPy and MnO_2_ nanoparticles [34]. The vapor-phase polymerization of PPy on MnO_2_ results in a unique hierarchical architecture where the clustered MnO_2_ nanostructures function as physical spacers, generating well-defined interparticle voids among polymerizing pyrrole units. This engineered morphology promotes the development of an extended conductive matrix during polymerization, as evidenced by the enhanced charge transfer kinetics. Notably, the conformal PPy coating establishes continuous conductive pathways that interconnect isolated MnO_2_ active centers, significantly reducing the average electron transport distance within the composite electrode [41]. Figure 6b,c display the CV profiles of MnO_2_/CC, PPy/CC, and PPy/MnO_2_/CC at varying scan rates (10–100 mV/s). All three electrodes show increasing current response with higher scan rates while maintaining their characteristic CV shapes, demonstrating excellent rate capability and capacitance retention [38].

The electrochemical storage characteristics were evaluated through galvanostatic charge–discharge measurements. Figure 6a shows the galvanostatic charge–discharge (GCD) curves of MnO_2_/CC, PPy/CC, and PPy/MnO_2_/CC at a current density of 2.5 mA/cm^2^. All electrodes exhibit quasi-triangular GCD profiles, indicating highly reversible charge–discharge behavior. The PPy/MnO_2_/CC hybrid composite shows markedly extended operational time windows during both charging and discharging phases. This pronounced increase in discharge duration directly reflects the composite’s enhanced charge storage capability [42,43]. The areal capacitance (*C*) of the electrodes was calculated using the following Equation [31]:

(4) $$C=\frac{I\;\Delta t}{S\;\Delta V}$$

where *I* is the discharge current (A), Δ*t* is the discharge time (s), *S* is the electrode area (cm^2^), and Δ*V* is the potential window (V). Based on this equation, the PPy/MnO_2_/CC electrode delivers an outstanding areal capacitance of 324.5 mF/cm^2^, significantly higher than those of MnO_2_/CC (123.1 mF/cm^2^) and PPy/CC (41.1 mF/cm^2^). The comparisons are listed in Table 1. We hold the opinion that the electrochemical performance enhancement of the MnO_2_/PPy/CC can be attributed to three key structural advantages: (1) the hierarchical porous architecture with folded nanostructures provides abundant active sites and facilitates electrolyte penetration, (2) the continuous conductive PPy network establishes efficient charge transfer pathways, and (3) the synergistic coupling between MnO_2_ and PPy optimizes both Faradaic and capacitive charge storage mechanisms. As expected and as shown in Figure 6b, the capacitance decreases with increasing current density, likely due to kinetic limitations in electrolyte ion diffusion [44]. Electrochemical impedance spectroscopy (EIS) was employed to investigate charge transfer dynamics. Figure 6c displays the Nyquist plots for MnO_2_/CC, PPy/CC, and PPy/MnO_2_/CC, with the inset showing an enlarged high-frequency region. All samples exhibit similar impedance spectra, featuring a semicircle in the high-frequency region (associated with charge transfer resistance, *R*ct) and a linear Warburg region at low frequencies (reflecting ion diffusion). The PPy/MnO_2_/CC composite shows the smallest semicircle diameter and the steepest Warburg slope, indicating enhanced charge transfer kinetics and superior capacitive behavior [20]. Additionally, as shown in the enlarged view of Figure 6c, the MnO_2_/PPy/CC electrode exhibits the lowest equivalent series resistance (2.15 Ω) compared to MnO_2_/CC (2.64 Ω) and PPy/CC (2.57 Ω), indicating superior electrical conductivity. The improved proton diffusion in PPy/MnO_2_/CC may be attributed to the conductive PPy network within the MnO_2_ matrix [45,46]. Cycling stability tests were conducted at 2.5 mA/cm^2^ for 5000 cycles (Figure 6d). The MnO_2_/CC and PPy/CC electrodes suffer significant capacitance losses (68% and 73%, respectively), whereas the PPy/MnO_2_/CC composite retains 91% of its initial capacitance. These results confirm that the PPy/MnO_2_/CC electrode displays comparable charge storage and cycling stability compared to the other electrodes, likely due to its larger effective surface area and more efficient electron transport pathways.

## 5. Conclusions

In summary, we successfully fabricated a novel MnO_2_/PPy composite electrode on carbon cloth via a combined hydrothermal and vapor-phase polymerization (VPP) approach. The synergistic interaction between MnO_2_ and PPy significantly enhances the electrochemical performance of the composite. The optimized electrode delivers a high areal capacitance of 324.5 mF/cm^2^ at 2.5 mA/cm^2^, along with excellent cycling stability, retaining 91% of its initial capacitance after 5000 cycles. These superior properties, coupled with the flexibility of the carbon cloth substrate, make the MnO_2_/PPy composite a promising candidate for next-generation flexible supercapacitors, particularly in wearable and smart electronic applications.

## Figures and Tables

**Table 1:** Comparison of the specific capacitance of the composite electrodes.

Electrodes	Fabrication Method	Capacitance	Current Density/Scan Rate	Ref.
MnO_2_/PPy/CC	Hydrothermal process	45.6 mF/cm^2^	2.5 mV/s	[47]
MnO_2_/PPy	In situ interfacial redox reaction	705 F/g	2 mV/s	[36]
MnO_2_/PPy	Layer-by-layer method	404 F/g	1 A/g	[46]
SWNTs@MnO_2_/PPy	Chemical vapor deposition	351 F/g	1 mV/s	[44]
MnO_2_/Ni(OH)_2_	One-pot method	1133.3 F/g	1 A/g	[48]
β-MnO_2_	Hydrothermal process	625 F/g	0.25 A/g	[49]
MnO_2_/PPy/CC	Vapor-phase polymerization	324.5 mF/cm^2^ (773 F/g)	2.5 mA/cm^2^	this work

## Data Availability

Data are contained within the article.

## References

[1] Lei S., Liu Y., Fei L., Song R., Lu W., Shu L., Mak C.L., Wang Y., Huang H. (2016). Commercial Dacron cloth supported Cu(OH)_2_ nanobelt arrays for wearable supercapacitors. J. Mater. Chem. A.

[2] Hu L.B., Pasta M., Mantia F.L., Cui L.F., Jeong S., Deshazer H.D., Choi J.W., Han S.M., Cui Y. (2010). Stretchable, Porous, and Conductive Energy Textiles. Nano Lett..

[3] Lu X., Yu M., Wang G., Tong Y., Li Y. (2014). Flexible solid-state supercapacitors: Design, fabrication and applications. Energy Environ. Sci..

[4] Zhao P., Yao M., Ren H., Wang N., Komarneni S. (2018). Nanocomposites of hierarchical ultrathin MnO_2_ nanosheets/hollow carbon nanofibers for high-performance asymmetric supercapacitors. Appl. Surf. Sci..

[5] Yu N., Yin H., Zhang W., Liu Y., Tang Z., Zhu M.Q. (2016). High-performance fiber-shaped all-solid-state asymmetric supercapacitors based on ultrathin MnO_2_ nanosheet/carbon fiber cathodes for wearable electronics. Adv. Energy Mater..

[6] Long X., Zeng Z., Guo E., Shi X., Zhou H., Wang X. (2016). Facile fabrication of all-solid-state flexible interdigitated MnO_2_ supercapacitor via in-situ catalytic solution route. J. Power Sources.

[7] Wang Y.M., Chen J.C., Cao J.Y., Liu Y., Zhou Y., Ouyang J.H., Jia D.C. (2014). Graphene/carbon black hybrid film for flexible and high rate performance supercapacitor. J. Power Sources.

[8] Abdelkader A.M., Karim N., Vallés C., Afroj S., Novoselov K.S., Yeates S.G. (2017). Ultraflexible and robust graphene supercapacitors printed on textiles for wearable electronics applications. 2D Mater..

[9] Xiong S., Zhang X., Chu J., Wang X., Zhang R., Gong M., Wu B. (2018). Hydrothermal Synthesis of Porous Sugarcane Bagasse Carbon/MnO_2_ Nanocomposite for Supercapacitor Application. J. Electron. Mater..

[10] Zhang X., Wang J., Liu J., Wu J., Chen H., Bi H. (2017). Design and preparation of a ternary composite of graphene oxide/carbon dots/polypyrrole for supercapacitor application: Importance and unique role of carbon dots. Carbon.

[11] Lee S., Nam G., Sun J., Lee J.S., Lee H.W., Chen W., Cho J., Cui Y. (2017). Enhanced Intrinsic Catalytic Activity of λ-MnO_2_ by Electrochemical Tuning and Oxygen Vacancy Generation. Angew. Chem. Int. Ed..

[12] El-Deab M.S., Ohsaka T. (2006). Manganese Oxide Nanoparticles Electrodeposited on Platinum Are Superior to Platinum for Oxygen Reduction. Angew. Chem. Int. Ed..

[13] Dong J., Lu G., Wu F., Xu C., Kang X., Cheng Z. (2018). Facile synthesis of a nitrogen-doped graphene flower-like MnO_2_ nanocomposite and its application in supercapacitors. Appl. Surf. Sci..

[14] Yu Z.N., Duong B., Abbitt D., Thomas J. (2013). Highly ordered MnO_2_ nanopillars for enhanced supercapacitor performance. Adv. Mater..

[15] Han X.L., Zhang J., Wang Z.S., Younus H.A., Wang D.W. (2024). Engineering the Microstructures of Manganese Dioxide Coupled with Oxygen Vacancies for Boosting Aqueous Ammonium-ion Storage in Hybrid Capacitors. Rare Met..

[16] Xu Z., Sun S., Cui W., Lv J., Geng Y., Li H., Deng J. (2018). Interconnected network of ultrafine MnO_2_ nanowires on carbon cloth with weed-like morphology for high-performance supercapacitor electrodes. Electrochim. Acta.

[17] Makgopa K., Ejikeme P.M., Jafta C.J., Raju K., Zeiger M., Presser V., Ozoemena K.I. (2015). A high-rate aqueous symmetric pseudocapacitor based on highly graphitized onion-like carbon/birnessite-type manganese oxide nanohybrids. J. Mater. Chem. A.

[18] Yun T.G., Hwang B.I., Kim D., Hyun S., Han S.M. (2015). Polypyrrole–MnO_2_-Coated Textile-Based Flexible-Stretchable Supercapacitor with High Electrochemical and Mechanical Reliability. ACS Appl. Mater. Interfaces.

[19] An J., Liu J., Ma Y., Li R., Li M., Yu M., Li S. (2012). Fabrication of graphene/polypyrrole nanotube/MnO_2_ nanotube composite and its supercapacitor application. Eur. Phys. J. Appl. Phys..

[20] Bahloul A., Nessark B., Briot E., Groult H., Mauger A., Zaghib K., Julien C.M. (2013). Polypyrrole-covered MnO_2_ as electrode material for supercapacitor. J. Power Sources.

[21] Sidhu N.K., Rastogi A.C. (2013). Nanoscale Blended MnO_2_ Nanoparticles in Electro-polymerized Polypyrrole Conducting Polymer for Energy Storage in Supercapacitors. MRS Proc..

[22] Shivakumara S., Munichandraiah N. (2019). In-situ preparation of nanostructured α-MnO_2_/polypyrrole hybrid composite electrode materials for high performance supercapacitors. J. Alloys Compd..

[23] Yalovega G.E., Brzhezinskaya M., Dmitriev V.O., Shmatko V.A., Ershov I.V., Ulyankina A.A., Chernysheva D.V., Smirnova N.V. (2024). Interfacial Interaction in MeOx/MWNTs (Me-Cu,Ni) Nanostructures as Efficient Electrode Materials for High-Performance Supercapacitors. Nanomaterials.

[24] Liu X., Guan C., Hu Y., Zhang L., Elshahawy A.M., Wang J. (2017). 2D Metal-Organic Frameworks Derived Nanocarbon Arrays for Substrate Enhancement in Flexible Supercapacitors. Small.

[25] Dubal D.P., Kim J.G., Kim Y., Holze R., Lokhande C.D., Kim W.B. (2014). Supercapacitors Based on Flexible Substrates: An Overview. Energy Technol..

[26] Wang W., Liu W., Zeng Y., Han Y., Yu M., Lu X., Tong Y. (2015). A Novel Exfoliation Strategy to Significantly Boost the Energy Storage Capability of Commercial Carbon Cloth. Adv. Mater..

[27] Zou N., Nie Q., Zhang X., Zhang G., Wang J., Zhang P. (2018). Electrothermal regeneration by Joule heat effect on carbon cloth based MnO_2_ catalyst for long-term formaldehyde removal. Chem. Eng. J..

[28] He X., Zhao Y., Chen R., Zhang H., Liu J., Liu Q., Song D., Li R., Wang J. (2018). Hierarchical FeCo_2_O_4_@polypyrrole core/shell nanowires on carbon cloth for high-performance flexible all-solid-state asymmetric supercapacitors. ACS Sustain. Chem. Eng..

[29] He S., Chen W. (2015). Application of biomass-derived flexible carbon cloth coated with MnO_2_ nanosheets in supercapacitors. J. Power Sources.

[30] Guo X., Bai N., Tian Y., Gai L. (2018). Free-standing reduced graphene oxide/polypyrrole films with enhanced electrochemical performance for flexible supercapacitors. J. Power Sources.

[31] Gupta S. (2008). Hydrogen bubble-assisted syntheses of polypyrrole micro/nanostructures using electrochemistry: Structural and physical property characterization. J. Raman Spectrosc..

[32] Sun Y., Jia D., Zhang A., Tian J., Zheng Y., Zhao W., Cui L., Liu J. (2019). Synthesis of polypyrrole coated melamine foam by in-situ interfacial polymerization method for highly compressible and flexible supercapacitor. J. Colloid Interface Sci..

[33] Rabchinskii M.K., Sysoev V., Ryzhkov S.A., Eliseyev I., Stolyarova D.Y., Antonov G.A., Struchkov N.S., Brzhezinskaya M., Kirilenko D.A., Pavlov S.I. (2022). A Blueprint for the Synthesis and Characterization of Thiolated Graphene. Nanomaterials.

[34] Fan X., Wang X., Li G., Yu A., Chen Z. (2016). High-performance flexible electrode based on electrodeposition of polypyrrole/MnO_2_ on carbon cloth for supercapacitors. J. Power Sources.

[35] Nagaraju G., Kakarla R., Cha S.M., Yu J.S. (2015). Highly flexible conductive fabrics with hierarchically nanostructured amorphous nickel tungsten tetraoxide for enhanced electrochemical energy storage. Nano Res..

[36] Wang J.G., Yang Y., Huang Z.H., Kang F. (2012). Rational synthesis of MnO_2_/conducting polypyrrole@carbon nanofiber triaxial nano-cables for high-performance supercapacitors. J. Mater. Chem..

[37] Lin Z., Xiang X., Chen K., Peng S., Jiang X., Hou L. (2019). Facile synthesis of MnO_2_ nanorods grown on porous carbon for supercapacitor with enhanced electrochemical performance. J. Colloid Interface Sci..

[38] Chen Y., Zhu X., Yang D., Wangyang P., Zeng B., Sun H. (2018). A novel design of poly (3,4-ethylenedioxythiophene):poly (styrenesulfonate)/molybdenum disulfide/poly (3,4-ethylenedioxythiophene) nanocomposites for fabric micro-supercapacitors with favourable performances. Electrochim. Acta.

[39] Wang Y., Huo W.C., Yuan X.Y., Zhang Y.X. (2020). Composite of Manganese Dioxide and Two-dimensional Materials Applied to Supercapacitors. Acta Phys. Chim. Sin..

[40] Zhou H., Yan Z., Yang X., Lv J., Kang L., Liu Z.H. (2016). RGO/MnO_2_/polypyrrole ternary film electrode for supercapacitor. Mater. Chem. Phys..

[41] Xue Y.J., Huo J.H., Wang X., Zhao Y.Z. (2024). ZnxMnO_2_/PPy Nanowires Composite as Cathode Material for Aqueous Zinc-Ion Hybrid Supercapacitors. Battery Energy.

[42] Mohd Abdah M.A.A., Mohammed Modawe Aldris Edris N., Kulandaivalu S., Abdul Rahman N., Sulaiman Y. (2018). Supercapacitor with superior electrochemical properties derived from symmetrical manganese oxide-carbon fiber coated with polypyrrole. Int. J. Hydrogen Energy.

[43] Ramli N.I.T., Abdul Rashid S., Sulaiman Y., Mamat M.S., Mohd Zobir S.A., Krishnan S. (2016). Physicochemical and electrochemical properties of carbon nanotube/graphite nanofiber hybrid nanocomposites for supercapacitor. J. Power Sources.

[44] Liang K., Gu T., Cao Z., Tang X., Hu W., Wei B. (2014). In situ synthesis of SWNTs@MnO_2_/polypyrrole hybrid film as binder-free supercapacitor electrode. Nano Energy.

[45] Zhang Z., Chi K., Xiao F., Wang S. (2015). Advanced solid-state asymmetric supercapacitors based on 3D graphene/MnO_2_ and graphene/polypyrrole hybrid architectures. J. Mater. Chem. A.

[46] Han G., Liu Y., Kan E., Tang J., Zhang L., Wang H., Tang W. (2014). Sandwich-structured MnO_2_/polypyrrole/reduced graphene oxide hybrid composites for high-performance supercapacitors. RSC Adv..

[47] Wang C., Zhan Y., Wu L.X., Li Y.Y., Liu J.P. (2014). High-voltage and High-rate Symmetric supercapacitor based on MnO_2_-polypyrrole hybrid nanofilm. Nanotechnology.

[48] Nie G.D., Zhang Z.Y., Liu Y.Q., Wang J., Fu C., Yin H.Q., Chen J., Zhao L., Pan Z. (2022). One-Pot Rational Deposition of Coaxial Double-Layer MnO_2_/Ni(OH)_2_ Nanosheets on Carbon Nanofibers for High-Performance Supercapacitors. Adv. Fiber Mater..

[49] Zhu S.J., Huo W.C., Wang T., Li K.L., Liu X.Y., Ji J.Y., Yao H., Dong F., Zhang Y., Zhang L. (2021). Compulsive malposition of birnessite slab in 2D-Parallel birnessite on β-MnO_2_ networks for enhanced pseudocapacitance performances. Nano Mater. Sci..

